# Metabolic Plasticity and Transcriptomic Reprogramming Orchestrate Hypoxia Adaptation in Yak

**DOI:** 10.3390/ani15142084

**Published:** 2025-07-15

**Authors:** Ci Huang, Yilie Liao, Wei Peng, Hai Xiang, Hui Wang, Jieqiong Ma, Zhixin Chai, Zhijuan Wu, Binglin Yue, Xin Cai, Jincheng Zhong, Jikun Wang

**Affiliations:** 1 Key Laboratory of Qinghai-Tibetan Plateau Animal Genetic Resource Reservation and Utilization, Ministry of Education and Sichuan Province, Key Laboratory for Animal Science of National Ethnic Affairs Commission, Southwest Minzu University, Chengdu 610041, China; huangci_ci@163.com (C.H.); wanghui892321@sina.cn (H.W.); majieqiong0059@163.com (J.M.); chaizhixin2525@163.com (Z.C.); wzjdream2005@163.com (Z.W.); yuebinglin123@163.com (B.Y.); caixin2323@126.com (X.C.); zhongjincheng518@126.com (J.Z.); 2 Zhongshan Institute for Drug Discovery (ZIDD), Shanghai Institute of Materia Medica, Chinese Academy of Sciences, Zhongshan 528400, China; liaoyilie329@zidd.ac.cn; 3 Key Laboratory of Animal Genetics and Breeding on Tibetan Plateau, Ministry of Agriculture and Rural Affairs, Qinghai University, Xining 810016, China; pengwei@qhu.edu.cn; 4 Guangdong Provincial Key Laboratory of Animal Molecular Design and Precise Breeding, School of Life Science and Engineering, Foshan University, Foshan 528225, China; vamyluo@126.com

**Keywords:** *Bos grunniens*, cardiac fibroblasts, hypoxia adaptation, transcriptome, gene expression

## Abstract

The yak (*Bos grunniens*) has exceptional hypoxia resilience, making it an ideal model for studying high-altitude adaptation. Here, we investigated the effects of oxygen concentration on yak cardiac fibroblast proliferation and the underlying molecular regulatory pathways using RNA sequencing (RNA-seq) and metabolic analyses. Decreased oxygen levels significantly inhibited cardiac fibroblast proliferation and activity. Intriguingly, while the mitochondrial DNA (mtDNA) content remained stable, we observed coordinated upregulation of mtDNA-encoded oxidative phosphorylation components. Live-cell metabolic assessment further demonstrated that hypoxia led to mitochondrial respiratory inhibition and enhanced glycolysis. RNA-seq analysis identified key hypoxia adaptation genes, including glycolysis regulators (e.g., *HK2*, *TPI1*), and hypoxia-inducible factor 1-alpha (*HIF-1α*), with Gene Ontology (GO) and Kyoto Encyclopedia of Genes and Genomes (KEGG) enrichment analyses highlighting their involvement in metabolic regulation. The protein–protein interaction network identified three consensus hub genes across five topological algorithms (*CCNA2*, *PLK1*, and *TP53*) that may be involved in hypoxia adaptation. These findings highlight the importance of metabolic reprogramming underlying yak adaptation to hypoxia, providing valuable molecular insights into the mechanisms underlying high-altitude survival.

## Simple Summary

Yaks are native to high-altitude environments and exhibit extraordinary physiological adaptations to hypoxia. Investigating their responses under hypoxic conditions could provide insights into the molecular mechanisms underlying their hypoxia tolerance. In this study, we systematically explored how different oxygen concentrations affect the proliferation and metabolism of yak cardiac fibroblasts. Our results revealed that hypoxia induces cell cycle arrest and reshapes energy metabolism by enhancing glycolysis and reducing mitochondrial ATP production. Transcriptomic analysis identified key hypoxia-responsive genes involved in metabolism that support cell proliferation and energy production, hypoxia-inducible factor 1 (HIF-1) signaling, and mitochondrial regulation. Protein interaction network analysis further highlighted several central genes, such as tumor protein p53 (TP53) and polo-like kinase 1 (PLK1), which may coordinate the hypoxic response. These findings suggest that metabolic reprogramming and core regulatory genes play critical roles in the cellular adaptation of yaks to hypoxic environments.

## 1. Introduction

Mitochondria are essential organelles with complex structures that play central roles in cellular energy metabolism, biological evolution, and speciation. Hypoxia, a major environmental stressor at high altitudes, affects cellular energy metabolism, primarily through mitochondrial function. Studies have shown that hypoxic exposure modulates mitochondrial enzyme activity to enhance oxygen utilization efficiency [1]. Mitochondrial responses to hypoxia involve changes in enzyme kinetics and dynamic regulation through mitochondrial fission, fusion, and mitophagy, which contribute to metabolic reprogramming and organelle quality control [2].

Among the mitochondrial components, NADH dehydrogenase genes (e.g., *NADH1*, *NADH2*, and *NADH5*) are crucial for oxidative phosphorylation and are frequently implicated in adaptation to hypoxia. For instance, genetic polymorphisms in *NADH1* and *NADH2* are associated with high-altitude adaptability in humans [3], and mutations in *NADH5* are associated with altered mitochondrial respiratory function in Tibetan chickens under hypoxic stress [4]. Additionally, positive selection signatures have been observed in mitochondrial genes such as *NADH2*, *NADH4*, and *ATPase6* in high-altitude birds [5]. Notably, increased mitochondrial DNA (mtDNA) copy number was associated with increased respiratory capacity [6], with significantly higher levels observed in human sperm samples collected at 5300 m than in those collected at 1400 m [7]. Studies in gastric cancer cells have further demonstrated that hypoxia reduces mitochondrial membrane potential and increases reactive oxygen species (ROS) levels and mtDNA copy numbers, leading to suppressed aerobic respiration [8]. Despite these insights, studies on mtDNA and mitochondrial adaptations in yaks are scarce.

Yaks are a vital livestock species for herders on the Qinghai–Tibet Plateau, providing essential resources such as meat, milk, fiber, and labor, and play a vital role in the livelihoods of local communities. An iconic species of high-altitude regions, yaks have evolved to withstand hypoxia, extreme temperature fluctuations, and harsh environmental conditions [9]. Their physiological adaptations include enhanced lung elasticity and capacity, larger hearts with a stronger myocardium [10], and increased lactate dehydrogenase activity in the liver, which promotes anaerobic metabolism. Furthermore, yaks exhibit increased mitochondrial density and vascularization in muscle tissues, which improve oxygen delivery and metabolic efficiency [11]. Despite these well-characterized physiological traits, the molecular mechanisms underlying mitochondrial plasticity in yak cells under hypoxic conditions remain largely unexplored, particularly in cardiac fibroblasts, which play a vital role in maintaining myocardial structure and function.

The heart and lungs are the key organs involved in transcriptional changes during hypoxia adaptation [12]. Transcriptome sequencing of yak and cattle heart tissues identified differentially expressed genes (DEGs), including *COX7C*, *NFATC1*, *MAPKAPK3*, *PIK3R5*, and *ATP7A*, suggesting their roles in the adaptation to hypoxia [13]. Studies on Tibetan yaks indicate that hypoxia adaptation is mediated through pathways such as phosphoinositide 3-kinase-Akt (PI3K-Akt) and hypoxia-inducible factor 1 (HIF-1) [14]. Mitochondrial oxidative phosphorylation (OXPHOS) and peroxisome genomes also exhibit strong selection under hypoxic conditions [15]. In Tibetan sheep, elevated altitude enhanced the reliance on PI3K-Akt and peroxisome proliferator-activated receptor (PPAR) signaling pathways, promoted ATP production, and alleviated hypoxia-induced stress [16].

Despite extensive research on hypoxia adaptation in humans, rodents, and birds, the molecular mechanisms underlying mitochondrial adaptation to hypoxia in yaks remain poorly understood [17,18]. Considering the pivotal role of mitochondria in energy homeostasis and hypoxic signaling, elucidating their function in yak cardiac fibroblasts may reveal important adaptive strategies. In this study, yak cardiac fibroblasts were used as a cellular model to investigate mitochondrial function under normoxic, hypoxic, and anaerobic conditions. Through transcriptome profiling, quantitative real-time PCR (qPCR), Seahorse metabolic analysis, and quantification of mtDNA copy number and cell viability, we aimed to delineate the aerobic and anaerobic metabolic capacities of yak cells. These insights will contribute to a deeper understanding of yak evolutionary adaptation and may inform genetic improvement strategies for high-altitude livestock, as well as medical approaches to hypoxia-related diseases.

Our findings not only expand the current understanding of mitochondrial adaptations in high-altitude mammals but also lay a foundation for translational applications in animal breeding and hypoxia-related disease management.

## 2. Materials and Methods

### 2.1. Animal Ethics

All animal experiments in this study were ethically reviewed by the Animal Care and Use Committee of Southwest Minzu University (licence number: S2023030068), and the entire experiment was conducted in strict accordance with the “Guidelines for the Care and Use of Laboratory Animals” promulgated by the Ministry of Science and Technology of China.

### 2.2. Isolation and Culture of Yak Cardiac Fibroblasts

Three healthy 12-month-old Maiwa yaks from Aba Tibetan Autonomous Prefecture were randomly selected as experimental animals. Approximately 6 mm^3^ of left ventricular heart tissue was excised, rinsed with sterile phosphate-buffered saline (PBS, Gibco; Thermo Fisher Scientific, Waltham, MA, USA) to remove residual blood, and immersed in a pre-prepared cryopreservation solution comprising 10% dimethyl sulfoxide (DMSO, Gibco; Thermo Fisher Scientific, Waltham, MA, USA), 10% fetal bovine serum (FBS, Gibco; Thermo Fisher Scientific, Waltham, MA, USA), and 80% complete culture medium (Gibco; Thermo Fisher Scientific, Waltham, MA, USA). The tissue samples were preserved in liquid nitrogen and delivered to the laboratory for subsequent experiments. Primary cardiac fibroblasts were cultured and isolated from heart tissue using the tissue adherence culture method, following the protocol described by Tian et al. [19].

### 2.3. Treatment of Yak Cardiac Fibroblasts Under Different Oxygen Concentrations

Third-passage cardiac fibroblasts derived from the three yaks were cultured to approximately 80% confluence and randomly assigned to three experimental groups. Cells in the hypoxia group were subjected to a hypoxic atmosphere (1% O_2_, 5% CO_2_, and 94% N_2_) for 24 h to simulate hypoxia. The anoxia group was treated with 1.5 μmol/L oligomycin A (Selleck Chemicals, Houston, TX, USA) for 20 min to inhibit mitochondrial oxidative phosphorylation and mimic anaerobic conditions. The control group was maintained under normoxic conditions (21% O_2_) for 24 h. Following treatment, cells from all groups were harvested for subsequent analyses.

### 2.4. Measurement of Yak Cardiac Fibroblast Viability Under Different Oxygen Conditions

The viability and proliferation of yak cardiac fibroblasts after oxygen treatment were assessed using a Cell Counting Kit-8 (CCK8) assay kit (MedChemExpress, Monmouth Junction, NJ, USA) according to the manufacturer’s protocol.

### 2.5. Respiration and Glycolysis Analyses

Approximately 1.8 × 10^4^ cells were seeded per well in an XF96 cell culture microplate (Seahorse Bioscience, North Billerica, MA, USA). The specific method is described in our previous study [20].

### 2.6. RNA Extraction and qPCR

Total RNA was isolated using TRIzol (Invitrogen, Carlsbad, CA, USA) and treated with a genomic DNA eraser (Takara, Dalian, China) to remove DNA contamination. First-strand complementary DNA (cDNA) was synthesized using the PrimeScript™ RT Reagent Kit (Takara, Dalian, China). qPCR was conducted using the SYBR^®^ Premix Ex TaqTM kit (Takara Bio Inc., Shiga, Japan). The gene-specific primers are listed in Supplementary Table S1.

### 2.7. Quantification of mtDNA Copy Number

The DNA extraction method by Tábara et al. [21] with mtDNA-specific primers (GenBank KR011113.1) and *GAPDH* as an internal reference gene (Supplementary Table S1) to avoid underestimating mtDNA abundance. The mtDNA copy number was calculated as the ratio of mtDNA to nuclear DNA (mtDNA/nDNA).

### 2.8. RNA Sequencing (RNA-Seq) of Yak Cardiac Fibroblasts

Yak cardiac fibroblasts treated with varying oxygen levels were sent to Beijing Novogene Biotechnology Co., Ltd. (Beijing, China) for library preparation and transcriptome sequencing on an Illumina platform. There were three biological replicates in each group. Raw data underwent quality control to obtain clean reads for bioinformatics analysis. The original messenger RNA (mRNA) sequencing data were submitted to the National Center for Biotechnology Information (NCBI) (PRJNA1106272).

### 2.9. Transcriptome Sequencing Data Processing and Bioinformatics Analysis

Raw RNA-seq reads were quality-checked using FastQC (v0.12.1) and trimmed with Trimmomatic (v0.39) to remove adapter sequences and low-quality bases. Clean reads were aligned to the Bos grunniens (Bosgru_v3.0 from Ensembl) reference genome using HISAT2 (2.2.1). The corresponding gene annotation file in GTF format was used to guide alignment and read counting.

Transcript assembly was performed using StringTie (v2.2.3) for each sample individually based on the aligned BAM files, producing a separate GTF annotation file per sample. These individual GTFs were then merged using the stringtie–merge function to create a unified transcriptome annotation (merged.gtf). The GTF file containing these novel genes was then merged with the reference GTF to generate a comprehensive annotation. Next, we used gffcompare (v0.12.5) to compare the merged GTF file against the reference GTF to identify novel transcripts. Transcripts that did not overlap with any known genes were classified as novel genes. This combined GTF file was used in subsequent quantification. Gene expression level quantification was performed using featureCounts (2.0.6), and expression levels were calculated as fragments per kilobase of transcript per million mapped reads (FPKM).

Differentially expressed genes (DEGs) were identified using the DESeq2 R package (1.42.0). Prior to DEG analysis, raw read counts were normalized to account for differences in sequencing depth across samples. A statistical model was then applied to estimate *p*-values for differential expression testing. Multiple testing correction was performed using the false discovery rate (FDR) method, and adjusted *p*-values (padj) were calculated. Genes with *p*-value ≤ 0.05 and |log_2_FoldChange| > 0 were considered significantly differentially expressed.

Gene Ontology (GO) and KEGG enrichment analyses were performed using the clusterProfiler R package (4.13.3) with the annotation database org.Bt.eg.db (*Bos taurus*). The enrichment results were simplified based on semantic similarity and visualized using the enrichplot (1.25.2.1) and ggplot2 (3.5.1) packages.

### 2.10. Protein–Protein Interaction (PPI) Network Analysis

To analyse protein–protein interactions (PPIs) among DEGs, all DEGs were mapped to the STRING database to identify potential interactions. An interaction confidence score threshold of ≥0.4 was set to construct the initial PPI network. The network was visualized using the Cytoscape bioinformatics platform (v3.10.1), and topological analysis was conducted to identify key genes. Specifically, the CytoHubba plugin was used to comprehensively evaluate node centrality, employing five algorithms—Degree, EPC, BottleNeck, MNC, and Stress—for multidimensional feature analysis. The Molecular Complex Detection (MCODE) plugin was used to detect the molecular complexes simultaneously. The parameters were set as follows: K-core = 2, node degree cutoff ≥ 2, maximum depth = 100, and node score cutoff = 0.2. Modules with scores of >4 were considered statistically significant. The results from both algorithms were integrated, and overlapping genes were identified to determine the final set of core hub genes.

### 2.11. Data Analysis

Data are presented as the mean ± standard error of the mean (SEM). Statistical significance was determined using an independent Student’s *t*-test for comparisons between two groups and one-way ANOVA for multiple group comparisons, followed by Duncan’s multiple range test for post hoc analysis. A *p*-value of <0.05 was considered statistically significant, with *, **, and *** denoting *p* < 0.05, *p* < 0.01, and *p* < 0.001, respectively. All statistical analyses and figure generation were performed using GraphPad Prism 8 (GraphPad Software, La Jolla, CA, USA). All the experiments were performed in triplicate.

## 3. Results

### 3.1. Hypoxia Inhibits Cell Proliferation

**Figure 1:**
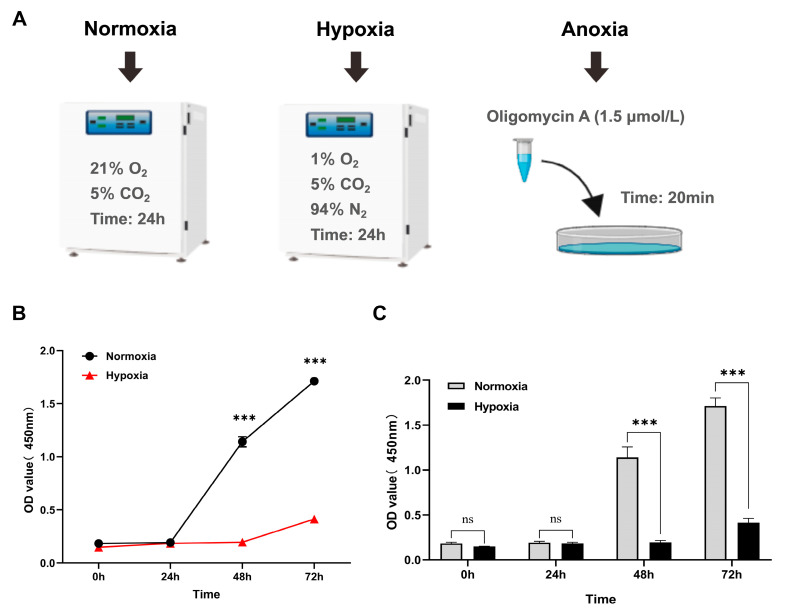
Effect of hypoxia on the proliferation activity of yak cardiac fibroblasts. (**A**) Schematic diagram of cell treatment under different oxygen concentrations. (**B**,**C**) The proliferation of cardiac fibroblasts cultured under normoxic and hypoxic conditions was evaluated by CCK8. OD450 values were recorded at 0, 24, 48, and 72 h. The results are expressed as mean ± SEM, and each group was repeated six times (*n* = 6). *** *p* < 0.001 and ns *p* > 0.05.

Cardiac fibroblast viability was measured using CCK8. The results of the CCK8 assay demonstrated that hypoxia significantly (*p <* 0.001) inhibits cell proliferation (Figure 1B,C).

### 3.2. Hypoxia Boosts Glycolysis but Limits Aerobic Energy Yield

**Figure 2:**
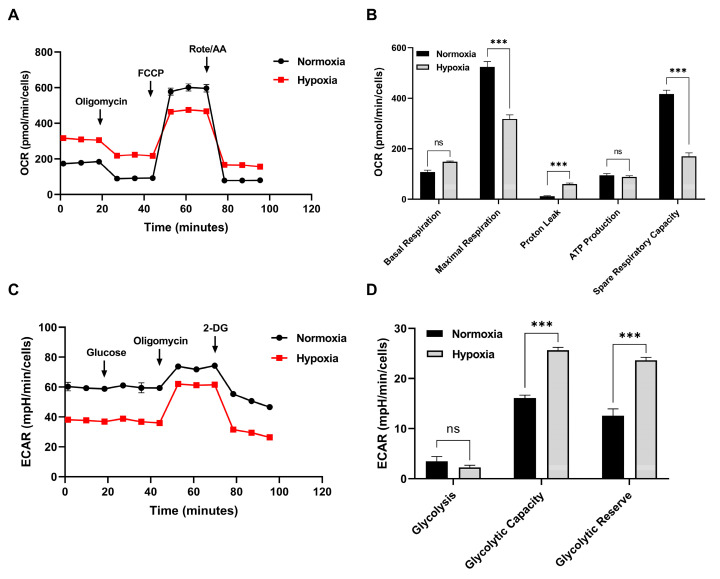
OCR and ECAR in yak cardiac fibroblasts under both normoxic and hypoxic conditions. (**A**,**B**) OCR assays: These panels display the OCR profiles, measured in pmol O_2_/min/cells. To determine specific respiratory parameters such as basal respiration, ATP turnover, proton leakage, and spare respiratory capacity, cells were sequentially treated with oligomycin, FCCP, and a combination of rotenone plus antimycin A. Non-mitochondrial respiration was then subtracted from total values. (**C**,**D**) ECAR assays: These panels show the ECAR profiles, expressed as mpH/min/cells. Basal glycolysis rates were established by measuring ECAR in the presence of glucose, while glycolytic reserve was assessed after oligomycin treatment. All data points represent the mean with SEM, based on six samples per group (*n* = 6). *** *p* < 0.001 and ns *p* > 0.05.

Hypoxia significantly increases extracellular acidification rate (ECAR) in yak cardiac fibroblasts, presenting enhanced glycolysis (glycolytic capacity and reserve both *p* < 0.001) (Figure 2C,D). Hypoxia resulted in a decrease in oxygen consumption (OCR) values and a decrease in maximal and spare respiratory capacity (*p* < 0.001). Despite this, basal aerobic respiration and ATP production remain similar to normoxic conditions (*p* > 0.05) (Figure 2A,B), suggesting yak cells maintain strong aerobic respiration even under hypoxia. However, proton leakage presented a significant increase (*p* < 0.001).

### 3.3. Hypoxia Upregulates Mitochondrial Coding Genes Without Changing MtDNA Levels

**Figure 3:**
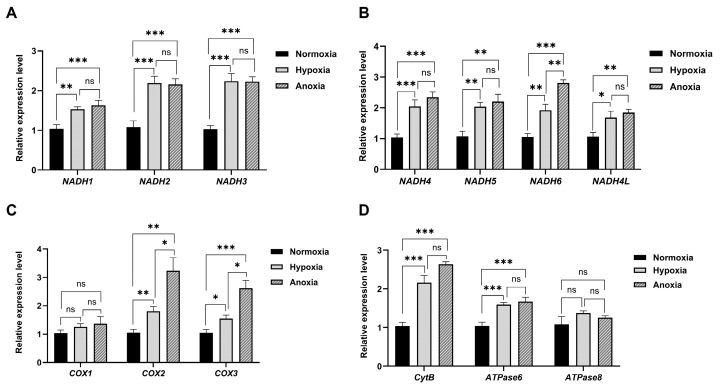
Relative expression of mitochondrial coding genes under normoxic, hypoxic, and anaerobic conditions is shown, with bars representing the SEM, *n* = 3 per group. (**A**,**B**) Relative expression levels of *NADH1*–*NADH4L* genes. (**C**) Relative expression levels of *COX1*–*COX3* genes. (**D**) Relative expression levels of *CytB*, *ATPase6*, and *ATPase8* genes. * *p* < 0.05, ** *p* < 0.01, *** *p* < 0.001, and ns *p* > 0.05.

The relative expression levels of the *NADH1*, *NADH2*, *NADH3*, *NADH4*, *NADH5*, *NADH6*, *NADH4L*, *CytB*, *ATPase6*, *ATPase8*, *COX1*, *COX2*, and *COX3* genes under normoxic, hypoxic, and anaerobic conditions are presented in Figure 3. As oxygen concentrations gradually decreased, the expression levels of most genes exhibited an increasing trend. Only the expression levels of the *NADH2*, *NADH3*, and *ATPase8* genes exhibited initial upregulation followed by a downward trend as the oxygen concentration decreased; however, this reduction did not reach statistical significance (*p* > 0.05). The expression levels of *NADH1*, *NADH4*, *NADH5*, *NADH4L*, *ATPase6*, and *CytB* significantly increased (*p* < 0.05) with decreasing oxygen concentrations, but the increase was not significant (*p* > 0.05) under hypoxic and anaerobic conditions. The expression levels of *NADH6*, *COX2*, and *COX3* increased with decreasing oxygen concentrations, and the differences in expression levels among all conditions were significant (*p* < 0.01). Overall, the gene expression levels of most genes changed significantly as the oxygen concentration decreased (*p* < 0.05).

MtDNA copy numbers in yak cardiac fibroblasts were quantified using qPCR under normoxic, hypoxic, and anaerobic conditions, expressed as the ratio of mtDNA to nuclear DNA (mtDNA/nDNA). Yak cardiac fibroblasts under normoxic, hypoxic, and anaerobic conditions carried equal mtDNA copy numbers (Supplementary Figure S1).

### 3.4. Transcriptome Sequencing Reveals Gene Expression Differences Under Different Oxygen Partial Pressures

Paired-end sequencing yielded averages of 6.75, 6.65, and 6.89 G raw bases per sample under normoxic, hypoxic, and anaerobic conditions, respectively, with 6.43, 6.35, and 6.43 G clean bases after quality control. Sequencing data quality was high, with a base error rate of 0.03%, Q20 scores above 96.99%, Q30 scores exceeding 91.73%, and GC content between 51.84% and 53.54%. Clean reads were mapped to the reference genome to determine their location, with mapping rates exceeding 92.43% (normoxia), 92.41% (hypoxia), and 93.12% (anoxia) for each condition (Supplementary Table S2). The reference genome was validated to be suitable for accurate assembly and annotation.

**Figure 4:**
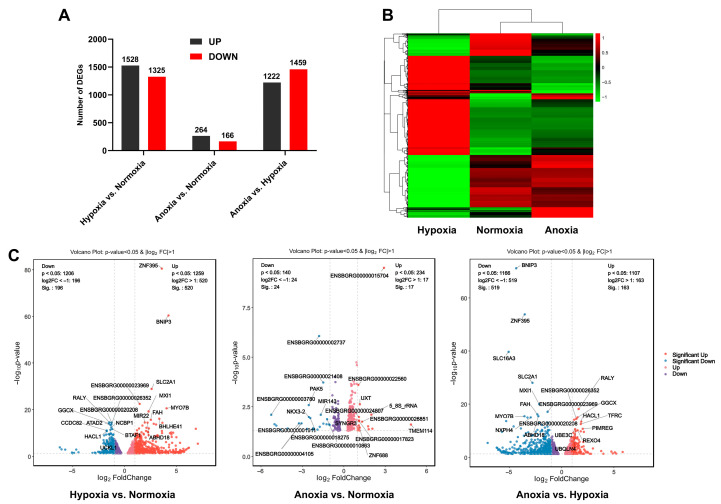
Differentially expressed gene (DEG) analysis of yak cardiac fibroblasts under anaerobic, hypoxic, and normoxic conditions. (**A**) Statistics of DEGs in the three groups. (**B**) Clustering map of DEGs. The horizontal axis represents the sample names, while the vertical axis shows the normalized FPKM values of the DEGs. Red indicates higher expression levels, whereas green indicates lower expression levels. (**C**) Volcano plots for the comparisons of hypoxia vs. normoxia, anoxia vs. normoxia, and anoxia vs. hypoxia, respectively.

Next, data were processed and analysed using the DESeq2 R package (v1.20.0). In total, 2853 DEGs were identified in the comparison between the hypoxia and normoxia groups, with 1528 upregulated genes (267 novel genes) and 1325 downregulated genes (119 novel genes) (Figure 4A and Supplementary Table S3). A total of 430 DEGs were identified when comparing the anoxia and normoxia groups, of which 264 were upregulated (30 novel genes) and 166 were downregulated (26 novel genes) (Figure 4A). In total, 2681 DEGs were identified in the comparison between the anoxia and hypoxia groups, with 1222 upregulated genes (115 novel genes) and 1459 downregulated genes (292 novel genes) (Figure 4A). A comparison of these data revealed that the hypoxia group exhibited the greatest number of DEGs relative to the normoxia group, with more genes upregulated than downregulated. The cluster diagram of DEGs (Figure 4B) demonstrates strong biological repeatability across the three conditions. The volcano plot results revealed that the hypoxia vs. normoxia and anoxia vs. hypoxia comparisons exhibited a greater number of significantly DEGs, including key genes such as *ZNF394*, *SL16A3*, *FAH*, *RALY*, and *BNIP3* (Figure 4C).

### 3.5. Functional Analysis of DEGs Revealed That Transcriptional Reprogramming Related to Nucleotide Synthesis, Carbon Metabolism, and Energy Production Plays a Critical Role in Yak Adaptation to Hypoxia

**Figure 5:**
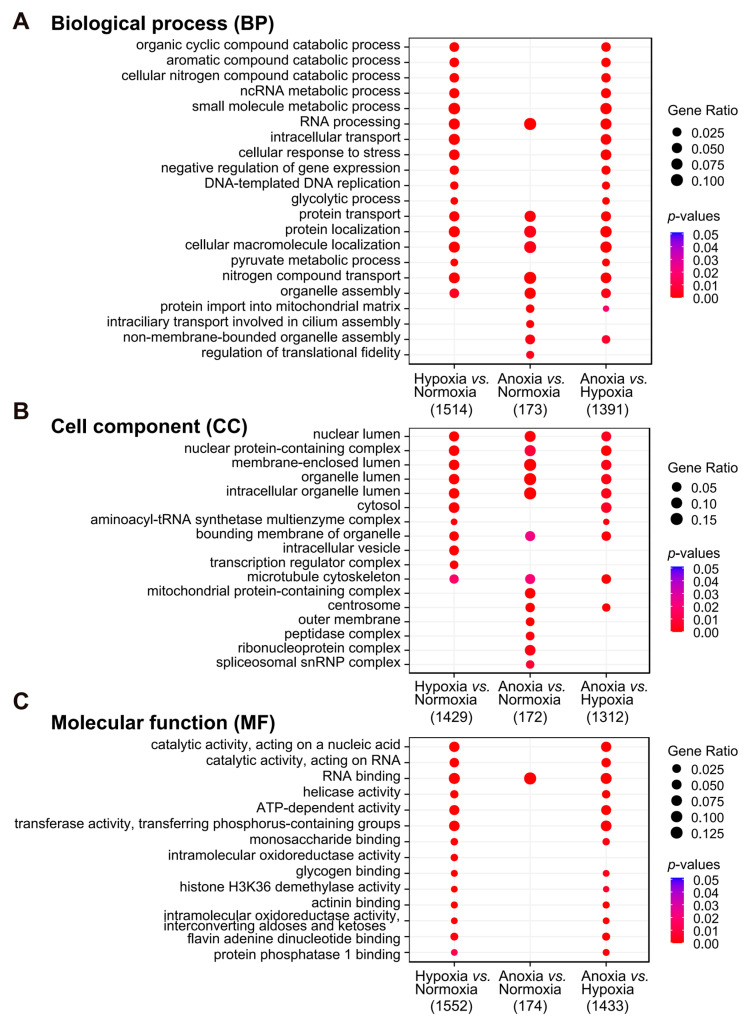
GO enrichment analysis. (**A**–**C**) Bubble charts showing the enriched pathways across the three GO categories. Redundant pathways were removed based on semantic similarity. The bubble size reflects the gene ratio, representing the proportion of DEGs in relation to the total genes assigned to a specific GO pathway. The color indicates the significance of the enrichment (*p*-values). The number of DEGs corresponding to each comparison in the overall enrichment analysis is annotated at the bottom of each panel.

Gene Ontology (GO) enrichment analyses revealed that the DEGs were primarily associated with biological processes such as nucleic acid (RNA/DNA) metabolism, response to stress, glycolytic processes, and protein transport and localization (Figure 5A and Supplementary Figure S2). These DEGs were mainly localized in the nucleus, transcription regulator complexes, cytoskeleton, and mitochondrial protein-containing complexes (Figure 5B and Supplementary Figure S2). They were involved in RNA binding, ATP-dependent activity, and intramolecular oxidoreductase activity, playing multiple functions such as binding, catalysis, and molecular transport (Figure 5C and Supplementary Figure S2). A large number of DEGs across the three groups were clustered in biological regulation, organelles, and macromolecular metabolism in cellular components, suggesting that mitochondria and glucose metabolism may play an important role in yak hypoxia adaptation.

**Figure 6:**
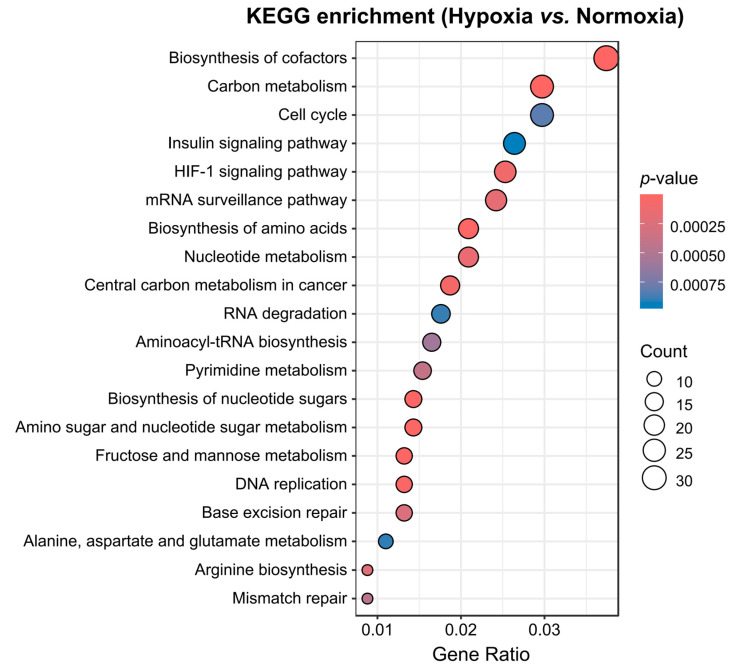
Dot plot showing KEGG enrichment analysis in the hypoxia and normoxia groups. The size of the dots represents the number of genes annotated to KEGG pathways. The color range from blue to red represents the significance of the enrichment. Gene ratio represents the ratio of the number of DEGs annotated to KEGG pathways to the total number of DEGs.

KEGG pathway enrichment analysis further highlighted pathways related to the cell cycle and metabolism, including carbon metabolism, the HIF-1 signaling pathway, and nucleotide and amino acid metabolism, which were significantly enriched in the hypoxia group compared with the normoxia group (Figure 6). Reactive oxygen species, oxidative phosphorylation, thermogenesis, and proteasomes were notably enriched in anoxia compared with normoxic conditions (Supplementary Figure S3A). Lipid metabolism and proteolysis were specifically enriched in the comparison between the anoxia and hypoxia groups (Supplementary Figure S3B), in addition to the shared pathways observed between the normoxia and hypoxia groups. These findings indicate that hypoxia acclimatization in yaks is closely related to metabolic remodeling.

### 3.6. PPI Network Construction

**Figure 7:**
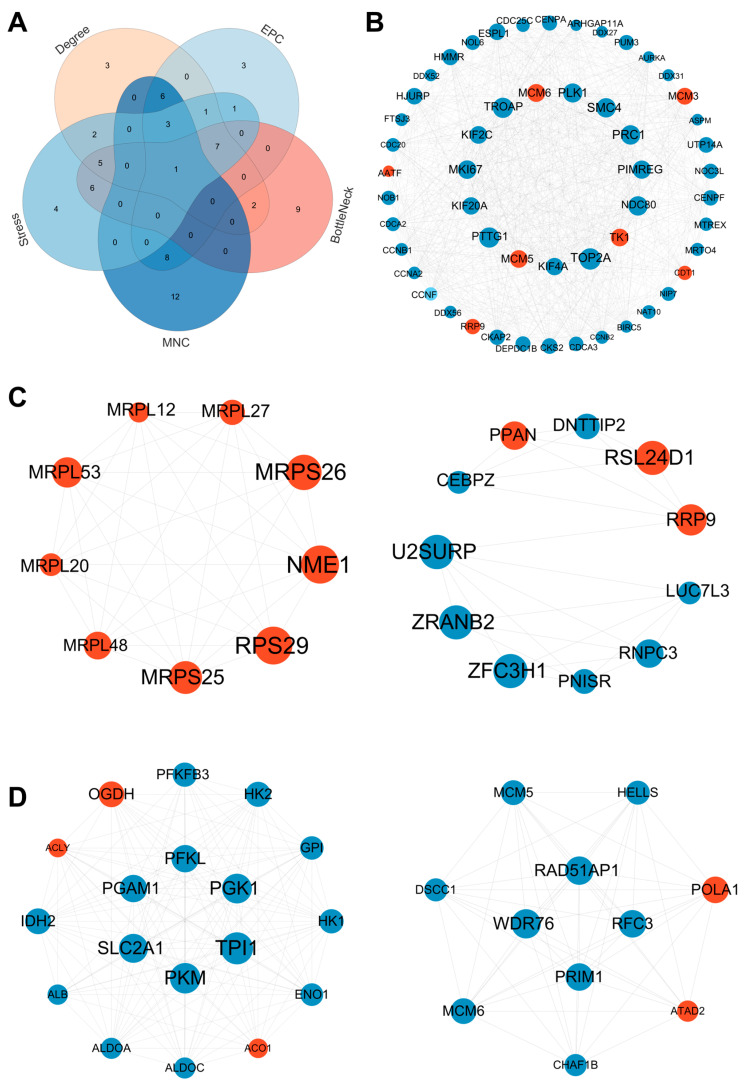
Identification of hub genes from the PPI network. (**A**) Venn diagram of hypoxia vs. normoxia. Venn diagram showing the overlap of the top 30 hub genes identified using five different CytoHubba algorithms (Degree, EPC, BottleNeck, MNC, and Stress). The numbers in the figure represent the quantity of genes identified. (**B**) PPI network diagrams of hypoxia vs. normoxia. (**C**) PPI network diagrams of anoxia vs. normoxia. (**D**) PPI network diagrams of anoxia vs. hypoxia. PPI network visualization of key genes constructed using STRING and Cytoscape (v3.10.1). Red nodes represent upregulated genes; blue nodes represent downregulated genes.

To further identify key hub genes, we mapped DEGs from three pairwise comparisons, hypoxia vs. normoxia, anoxia vs. normoxia, and anoxia vs. hypoxia, to the STRING database to evaluate PPI relationships and construct corresponding PPI networks. First, in the comparison between hypoxic and normoxic conditions, we used five algorithms in the CytoHubba plugin of Cytoscape (v3.10.1) to rank the top 30 hub genes in the PPI network. The Venn analysis of the results from each algorithm revealed one overlapping hub gene: *CCNA2* (Figure 7A). The MCODE plugin identified three tightly connected modules consisting of 80 DEGs and 851 interaction pairs (Figure 7B and Supplementary Figure S4C). The Venn analysis confirmed that one hub gene was included in the MCODE-identified module. Second, for the comparison between anoxia and normoxia, we used CytoHubba to identify the top 30 hub genes and obtained seven overlapping hub genes across the five algorithms: *ATP5F1D*, *NDUFS4*, *NDUFV1*, *PSMB6*, *RPS29*, *RRP9*, and *UQCR10* (Supplementary Figure S4A). MCODE analysis revealed two modules comprising 20 DEGs and 60 interaction pairs (Figure 7C), with two hub genes (*RPS29* and *RRP9*) present in the MCODE-derived modules. Finally, in a comparison between the anoxic and hypoxic conditions, CytoHubba identified seven overlapping hub genes: *ALB*, *CASP3*, *EZH2*, *H3F3A*, *MRTO4*, *PLK1*, and *TP53* (Supplementary Figure S4B). The MCODE analysis detected three modules containing 77 DEGs and 695 interaction pairs (Figure 7D and Supplementary Figure S4D). *ALB*, *MRTO4*, and *PLK1* were also found among the CytoHubba-identified hub genes.

### 3.7. qPCR Confirms High Consistency and Reproducibility with RNA-Seq Data

**Figure 8:**
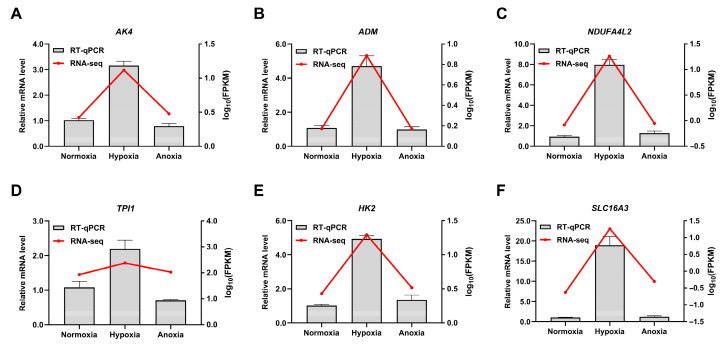
Validation of differentially expressed genes. (**A**–**F**) represent the relative expression levels and RNA-seq expression trends of the genes *AK4*, *ADM*, *NDUFA4L2*, *TPI1*, *HK2,* and *SLC16A3* under normoxic, hypoxic and anaerobic conditions, respectively.

Furthermore, six DEGs (*AK4*, *ADM*, *HK2*, *TPI1*, *NDUFA4L2*, and *SLC16A3*) were randomly selected for validation using qPCR. These data showed that the expression patterns of the two methods were highly consistent (Figure 8) and demonstrated a strong positive correlation, with a correlation coefficient above 0.5, normalized to the housekeeping gene *GAPDH*. The qPCR results confirmed the high reproducibility and reliability of the gene expression profiling in our study.

## 4. Discussion

Mitochondria are central to cellular energy metabolism and adaptation, as they regulate oxidative phosphorylation, ATP synthesis, and redox balance. Under hypoxia, mitochondria optimize electron transport chain efficiency, enhance glycolysis, and modulate oxidative phosphorylation to sustain cell function [22]. However, the specific mitochondrial mechanisms contributing to hypoxia tolerance in high-altitude species, such as yaks, remain unclear. This study assessed the cardiac fibroblast proliferation, mitochondrial gene expression, mtDNA copy number, OCR, ECAR, and transcriptomic changes at different oxygen levels to elucidate the role of mitochondria in yak hypoxia adaptation.

Yak cardiac fibroblasts display a coordinated metabolic adaptation to hypoxia that prioritizes energy conservation and survival over growth. One of the most notable responses was the suppression of cell proliferation, a process that consumes substantial energy. Under limited oxygen availability, reducing proliferative activity likely serves as an energy-saving strategy to conserve ATP, helping cells redirect resources toward essential maintenance and stress responses [23].

MtDNA copy number remained unchanged under hypoxic conditions, consistent with findings in human lymphocytes [24] but contrasting with the decrease observed in hypoxic trophoblasts [25]. These discrepancies may reflect species or cell-type-specific responses, underscoring the unique capacity of yaks to maintain mitochondrial genomic stability under hypoxic stress. However, we observed a significant upregulation of mtDNA-encoded oxidative phosphorylation (OXPHOS) genes, including *NADH1*, *NADH2*, *NADH3*, *NADH4*, *NADH5*, *NADH6*, *NADH4L*, *CytB*, *COX2*, *COX3*, and *ATPase6*. Previous studies have linked *NADH1* and *NADH2* to hypoxia adaptation via the modulation of NADH dehydrogenase activity [3,26], and ATP6/8 to improved oxygen utilization [27]. This selective transcriptional upregulation suggests a functional remodeling of the electron transport chain (ETC) subunits to preserve mitochondrial capacity under stress, rather than an increase in mitochondrial biogenesis.

However, the Seahorse XF analysis indicated a significant decline in mitochondrial functional parameters, including maximal respiration and spare respiratory capacity. This suggests that although OXPHOS gene expression increases, it does not fully translate into enhanced respiratory capacity, potentially reflecting a strategic metabolic downregulation to reduce oxygen demand and limit oxidative stress. Concurrently, we observed a marked increase in mitochondrial proton leakage, which, though energetically inefficient, serves as a protective mechanism by lowering mitochondrial membrane potential, reducing ROS production, and supporting non-shivering thermogenesis—critical under cold and hypoxic conditions [28,29]. This phenomenon has also been observed in rats under simulated high-altitude conditions, where uncoupling protein 4 and 5 (UCP4/5) expression and proton leakage increased significantly [30], and in yak primary cells, which exhibited greater proton leakage and glycolytic capacity than native beef cattle [20]. Glycolytic capacity and reserve were significantly enhanced to compensate for diminished oxidative phosphorylation, consistent with previous findings [31]. This metabolic shift toward glycolysis—often described as the Pasteur effect—provides a rapid, oxygen-independent ATP source, ensuring energy supply despite compromised mitochondrial respiration.

Building on our findings of mitochondrial gene expression remodeling and metabolic reprogramming in yak cardiac fibroblasts under hypoxia, we expanded our analysis to investigate transcriptomic changes in yak cardiac fibroblasts under varying oxygen levels. Only a few DEGs were detected 20 min after oligomycin A treatment in yak cardiac fibroblasts, although longer exposure caused significant cell death. As an ATP synthase inhibitor, oligomycin A rapidly disrupts oxidative phosphorylation and induces energy stress; however, transcriptomic responses typically require more time for signaling, transcription factor activation, and chromatin remodeling [32]. Early stress responses often depend on post-translational regulation, metabolic reprogramming, and selective translation rather than on new transcription [33,34]. For instance, oligomycin can quickly activate the PERK-eIF2α pathway, suppress global translation, and enhance ATF4 synthesis without requiring transcription [35]. Even in macrophages, a 4 h treatment with oligomycin induces few DEGs [36], while broader transcriptomic responses to mitochondrial stress, such as hypoxia, usually emerge after 2–4 h [37]. These findings suggest that yak cardiac fibroblasts rely primarily on non-transcriptional mechanisms to adapt to acute mitochondrial stress.

RNA-seq revealed the greatest number of DEGs under hypoxia and normoxia (2853), indicating a strong cellular response consistent with the transcriptomic changes reported in cattle lungs exposed to altitude stress [38]. GO and KEGG analyses showed that these DEGs were enriched in cell cycle, oxidative phosphorylation, carbon metabolism, ATP-dependent activity, mitochondrial function, and the HIF-1 signaling pathway. The upregulation of key glycolytic enzymes (PGK1, HK2, and PGAM1) under hypoxia, driven by HIF-1α, further supports the metabolic shift towards glycolysis to sustain ATP production [39,40]. HIF-1α directly induces adrenomedullin (ADM) to inhibit apoptosis and promote angiogenesis and cell survival. It also upregulates glycolytic enzymes such as hexokinase 2 (HK2), boosting anaerobic metabolism while suppressing mitochondrial respiration to limit ROS production [41]. NDUFA4L2, another HIF-1α target, inhibits mitochondrial complex activity, reducing oxygen consumption and ROS generation [42]. Additionally, HIF-1α enhances glycolysis by modulating enzymes such as PFKL, PDK1, and TPI1, further optimising energy metabolism under hypoxic conditions [43]. These findings suggest that yaks adapt to hypoxia not by increasing mitochondrial biogenesis but through transcriptional reprogramming of mitochondrial genes and metabolic pathways, ensuring efficient ATP production and thermogenic support in extreme environments.

PPI network analysis revealed that both CytoHubba and MCODE plugins effectively identified core hub genes across all three comparisons, validating their importance under varying oxygen conditions. Although no common hub genes were identified across the three comparison groups, *CCNA2*, *PLK1*, and *TP53* may serve as key regulatory factors in the response of cardiac fibroblasts to changes in oxygen concentration. The *TP53* gene encodes the p53 protein, a key tumor suppressor known as the “guardian of the genome” for its central role in maintaining genomic stability. As a transcription factor, p53 regulates DNA repair, cell cycle arrest, apoptosis, autophagy, and senescence [44]. In hypoxic environments, p53 activity is modulated to manage oxidative stress and preserve cellular homeostasis. Notably, specific *TP53* mutations may offer adaptive advantages in cold or hypoxic conditions [45]. Therefore, *TP53* may contribute to the hypoxia adaptation of yak cardiac fibroblasts by coordinating DNA repair, cell cycle regulation, and apoptosis. In human and mouse cells, hypoxia induces cell cycle arrest by downregulating genes such as *CCNA2* via HIF-1 signaling pathway [46]. A similar mechanism may operate in yaks, where reduced proliferation under hypoxia helps conserve energy and maintain cellular stability. *PLK1*, upregulated via HIF-dependent pathways, supports cell cycle progression and survival under oxygen-deprived conditions [47]. The overlap of these genes across multiple network analysis algorithms underscores their central role in cell cycle regulation, metabolic adaptation, and protein quality control under high-altitude hypoxia.

Collectively, these results support a model whereby yak cardiac fibroblasts reprogram their metabolism in response to hypoxia through five key strategies: (1) proliferation inhibition to conserve energy; (2) enhanced glycolytic flux to sustain ATP production oxygen-independently; (3) suppression of mitochondrial respiration to mitigate hypoxic stress and damage; (4) increased proton leakage to reduce ROS and support thermogenesis; (5) compensatory upregulation of OXPHOS genes expression to maintain essential ETC function.

Although this study provides valuable insights into the hypoxia adaptation mechanisms of yak cardiac fibroblasts, it has several limitations. First, while transcriptomic and metabolic profiling revealed key pathways and hub genes, functional validation (e.g., gene knockdown or overexpression) was not performed to confirm their specific roles. Second, the short-term oligomycin A treatment used for mitochondrial stress analysis may not have been sufficient to elicit a full transcriptional response. Future studies should include functional assays, integrative omics analyses, and mechanistic validation to further elucidate the molecular mechanisms underlying yak adaptation to high-altitude hypoxia.

## 5. Conclusions

This study revealed the adaptive mechanisms of yak cardiac fibroblasts to hypoxia, showing that hypoxia inhibits cell proliferation, causes mitochondrial damage, and disrupts energy metabolism. Despite stable mtDNA copy numbers, oxidative phosphorylation-related genes (*NADH1*, *NADH2*, *NADH4*, *NADH5*, *NADH4L*, *CytB*, *ATPase6*, *COX1*, *COX2*, and *COX3*) were significantly upregulated, suggesting an optimized respiratory chain for adaptation to hypoxia. Seahorse XF analysis indicated reduced oxidative phosphorylation, enhanced glycolytic potential, and increased proton leakage, possibly aiding non-shivering thermogenesis. Transcriptome analysis highlighted key pathways related to cell cycle and metabolism, including carbon metabolism, HIF-1 signaling pathway, and nucleotide and amino acid metabolism, involving critical genes (*AK4*, *SLC16A3*, *PGK1*, *HK2, PGAM1*, *PDK1*, *TPI1*, *CCNA2*, *PLK1*, and *TP53*). These findings suggest that yaks adapt to high-altitude hypoxia by regulating glycolysis and mitochondrial function to optimise energy metabolism and enhance antioxidant capacity. These findings provide fundamental data for further exploration of hypoxia adaptation mechanisms in yaks and are beneficial for exploring and utilising yak genetic resources.

## Data Availability

All data generated or analyzed during this study are included in this published article.

## Supplementary Materials

The following supporting information can be downloaded at: https://www.mdpi.com/article/10.3390/ani15142084/s1, Table S1: Primer sequence information; Table S2: Quality assessment of transcriptome sequencing data under normoxic, hypoxic and anaerobic conditions; Table S3: Number of differentially expressed genes; Figure S1: Measurement of mtDNA copy number in yak cardiac fibroblasts under normoxic, hypoxic and anaerobic conditions; Figure S2: Gene Ontology (GO) enrichment analysis; Figure S3: Kyoto Encyclopedia of Genes and Genomes (KEGG) enrichment analysis; Figure S4: Identification of hub genes from the PPI network.

## Abbreviations

The following abbreviations are used in this manuscript:

MtDNA	Mitochondrial DNA
ROS	Reactive oxygen species
DEGs	Differentially expressed genes
PI3K-Akt	Phosphoinositide 3-kinase–Akt
HIF-1	Hypoxia-inducible factor 1
PPAR	Peroxisome proliferator-activated receptor
qPCR	Quantitative real-time PCR
HIF-1α	Hypoxia-inducible factor 1-alpha
PBS	Phosphate-buffered saline
DMSO	Dimethyl sulfoxide
FBS	Fetal bovine serum
CCK8	Cell counting kit-8
NCBI	National Center for Biotechnology Information
RNA-seq	RNA sequencing
mRNA	Messenger RNA
KEGG	Kyoto Encyclopedia of Genes and Genomes
PPI	Protein–protein interaction
MCODE	Molecular complex detection
SEM	Standard error of the mean
ECAR	Extracellular acidification rate
OCR	Oxygen consumption rate
DEG	Differentially expressed gene
GO	Gene Ontology
OXPHOS	Oxidative phosphorylation
ETC	Electron transport chain
