# Supplementary material for: Metabolic Plasticity and Transcriptomic Reprogramming Orchestrate Hypoxia Adaptation in Yak
Source: Animals (Basel). 2025 Jul 15;15(14):2084. doi: 10.3390/ani15142084 (PMC12291710; doi:10.3390/ani15142084)
Supplement: Supplementary file 1 [file animals-15-02084-s001.zip › animals-3710932-supplementary.pdf]

animals

MDPI

Supplementary Information

Metabolic plasticity and transcriptomic reprogramming orchestrate hypoxia adaptation in yak

Ci Huang[1], Yilie Liao[2], Wei Peng[3], Hai Xiang[4], Hui Wang[1], Jieqiong Ma[1], Zhixin Chai[1], Zhijuan Wu[1], Binglin Yue[1], Xin Cai[1], Jincheng Zhong[1], and Jikun Wang[1,*]

Supplementary Tables

Table S1. Primer sequence information.

	Primer sequence (5′–3′)	Tm/℃	Product size/bp	Application
AK4	F：ACATCAAGGCCAACACGGAA	59	115	qPCR
	R：CGCCTGTTCTCCAACTCCAA			
ADM	F：TGGTTCCCGTCGCCCTCCTGTA	59	107	qPCR
	R：AGCCCACTTATTCCATTTCTTTCG			
HK2	F：AAATGGAGCGAGGTCTGAGC	59	144	qPCR
	R：GCAGGACCCGGAAATTGGTA			
NDUFA4L2	F：TCTGGTGGGACCGAAAGAAC	58	246	qPCR
	R：CCGGCTGTGTAAGCAGAAAC			
SLC16A3	F：ATGGTGTCTGCGTCCTTCTG	58	107	qPCR
	R：AGCATGATGAGTGAGGGCTG			
TPI1	F：ACTGGAAGATGAACGGGAGGAA	59	119	qPCR
	R：GAAGTCAATGTAGGCGGTGGG			
GAPDH	F：GTGATGCTGGTGCTGAGTAT	59	143	qPCR
	R：GCTCTCACATTCCTAAGTCC			
NADH1	F：TCCTATCTTATTAGCCGTAGCATTC	59	80	qPCR
	R：TTGGACCTTTTCGGAGTTGTAT			
NADH2	F：GAGTCCCAGAAGTAACGCAAGG	60	171	qPCR
	R：TTAGTCCTCCTCAGCCTCCAAT			
NADH3	F：TGCTTACCATAGCCCTCTTCC	59	86	qPCR
	R：TTCGGTTCATTCCAGTCCTTT			
NADH4	F：ACACCTTGAAGTTACATAGGAGCA	58	109	qPCR
	R：TTCGGCTGTGGATTCGTTC			
NADH5	F：CCTCATTGTTGGCAGTCTCG	58	114	qPCR
	R：AAAAGGGCTCAGGCGTTG			
NADH6	F：AATCCCTATGGCCTCCTCACT	59	158	qPCR
	R：GGGCATTTATTACTGGCTTGTTG			
NADH4L	F：TTCGCAGCCTGTGAAGCA	60	99	qPCR
	R: GCATTGGAGTAGGTTGAGATTTTGT			
ATPase6	F：CTGTGAGCAGGAGCCGTAAT	60	170	qPCR
	R：ATGTTGGCTGTTAACCGCAC			
ATPase8	F：AGACACGTCAACATGACTGACA	58	158	qPCR
	R：TCGTTCATTTTGTTTCTCAAGGGG			
cytB	F：ATCATTTTGAGGGGCAACAGT	58	141	qPCR
	R：AGGATAAAGTGGAAAGCGAAGA			
COX1	F：TTACAATGCTGCTAACAGACCG	58	105	qPCR

	R：GGTGCCCAAAGAATCAGAA-TAAGT			
COX2	F：GCCGTCTGAACCAAACAACC<br>R：CAAGGACGATGGGCATAAAA	58	105	qPCR
COX3	F：ACTTTCCAAGGGCACCATACC<br>R：GCAGTGGGACTTCTAGGGGAT	59	199	qPCR
β-actin	F：TGATGATATTGCTGCGCTCG<br>R：TACGAGTCCTTCTGGCCCAT	57	153	qPCR
mtDNA	F：AATCCTACAAATCCTCACAGG<br>R：TTGAAGCTCCGTTTGCGTGT	60	139	qPCR

Table S2. Quality assessment of transcriptome sequencing data under normoxic, hypoxic and anaerobic conditions.

Sample	Raw reads	Raw bases	Clean reads	Clean bases	Error rate	Q20（%）	Q30（%）	GC content（%）	Total map（%）
Normoxia 1	45386830	6.81G	41216782	6.18G	0.03	97.6	93.29	52.97	92.77%
Normoxia 2	46860072	7.03G	45787684	6.87G	0.03	97.24	92.36	51.84	92.43%
Normoxia 3	42756852	6.41G	41520648	6.23G	0.03	97.42	92.98	52.64	92.45%
Hypoxia 1	43182814	6.48G	42142780	6.32G	0.03	97.6	93.18	52.62	92.75%
Hypoxia 2	46074900	6.91G	44676008	6.7G	0.03	96.99	91.73	52.35	92.41%
Hypoxia 3	43803876	6.57G	40139150	6.02G	0.03	97.71	93.59	53.49	93.23%
Anoxia 1	45512586	6.83G	44116682	6.62G	0.03	97.84	93.86	52.98	94.27%
Anoxia 2	45941254	6.89G	41908932	6.29G	0.03	97.64	93.33	53.3	93.28%
Anoxia 3	46341612	6.95G	42626782	6.39G	0.03	97.15	92.33	53.54	93.12%

Table S3. Number of differentially expressed genes.

DEG sets	All DEGs	Upregulated	Downregulated
Anoxia vs. Hypoxia	2681	1222	1459
Anoxia vs. Normoxia	430	264	166
Hypoxia vs. Normoxia	2853	1528	1325

animals

Supplementary Figures

Figure S1. Measurement of mtDNA copy number in yak cardiac fibroblasts under normoxic, hypoxic and anaerobic conditions.

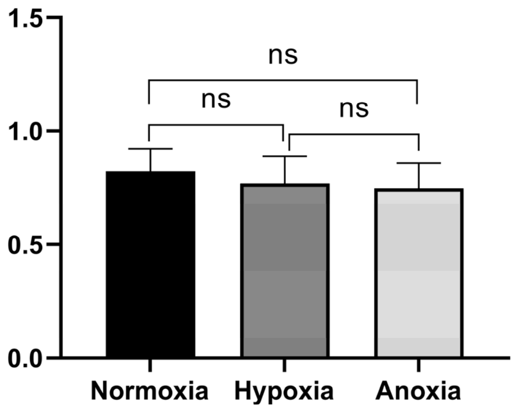

Bars represent the standard error of the mean (SEM), n = 3 per group. *$p < 0.05$, **$p < 0.01$, ***$p < 0.001$ and ns $p > 0.05$.

Figure S2. Gene Ontology (GO) enrichment analysis

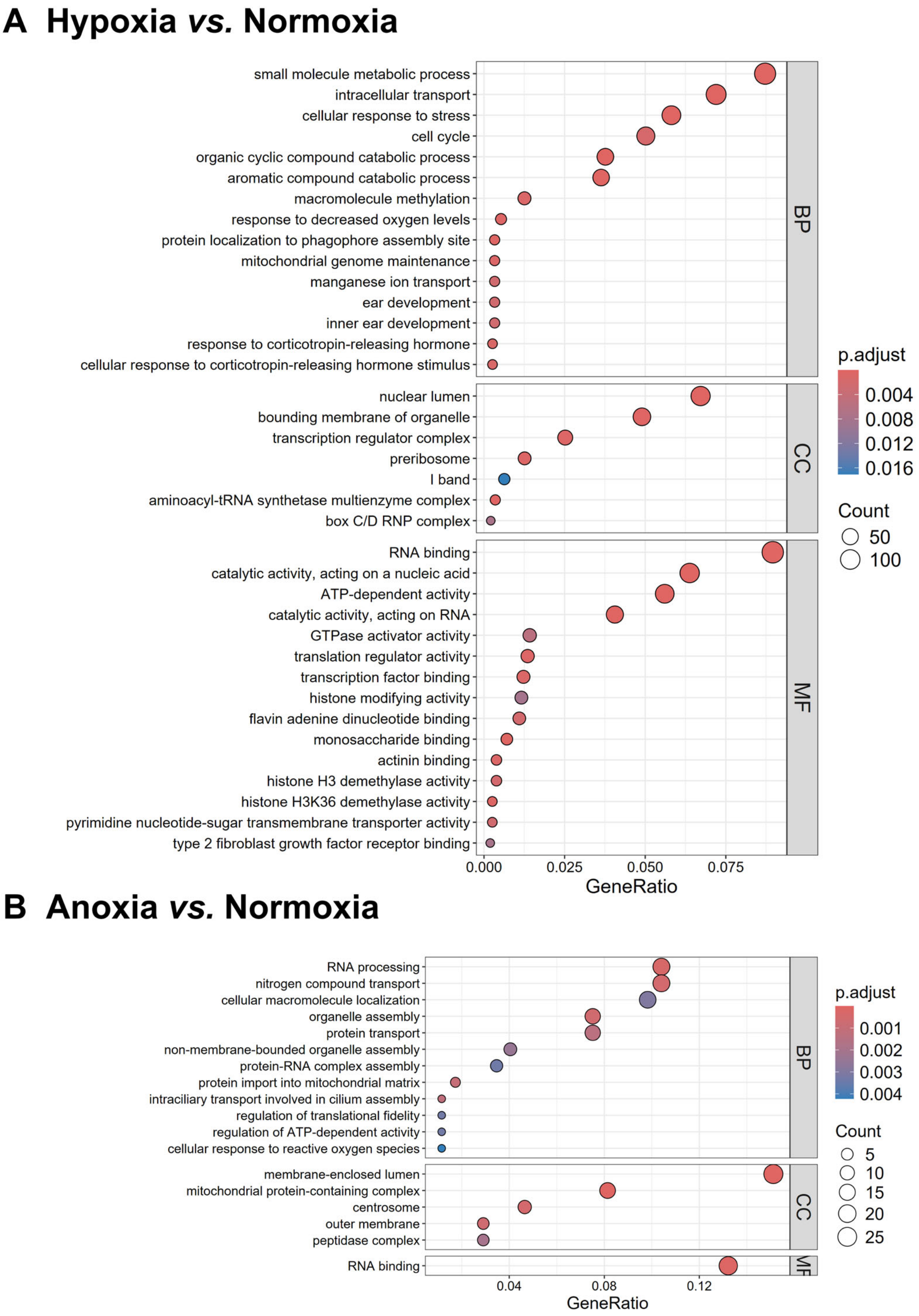

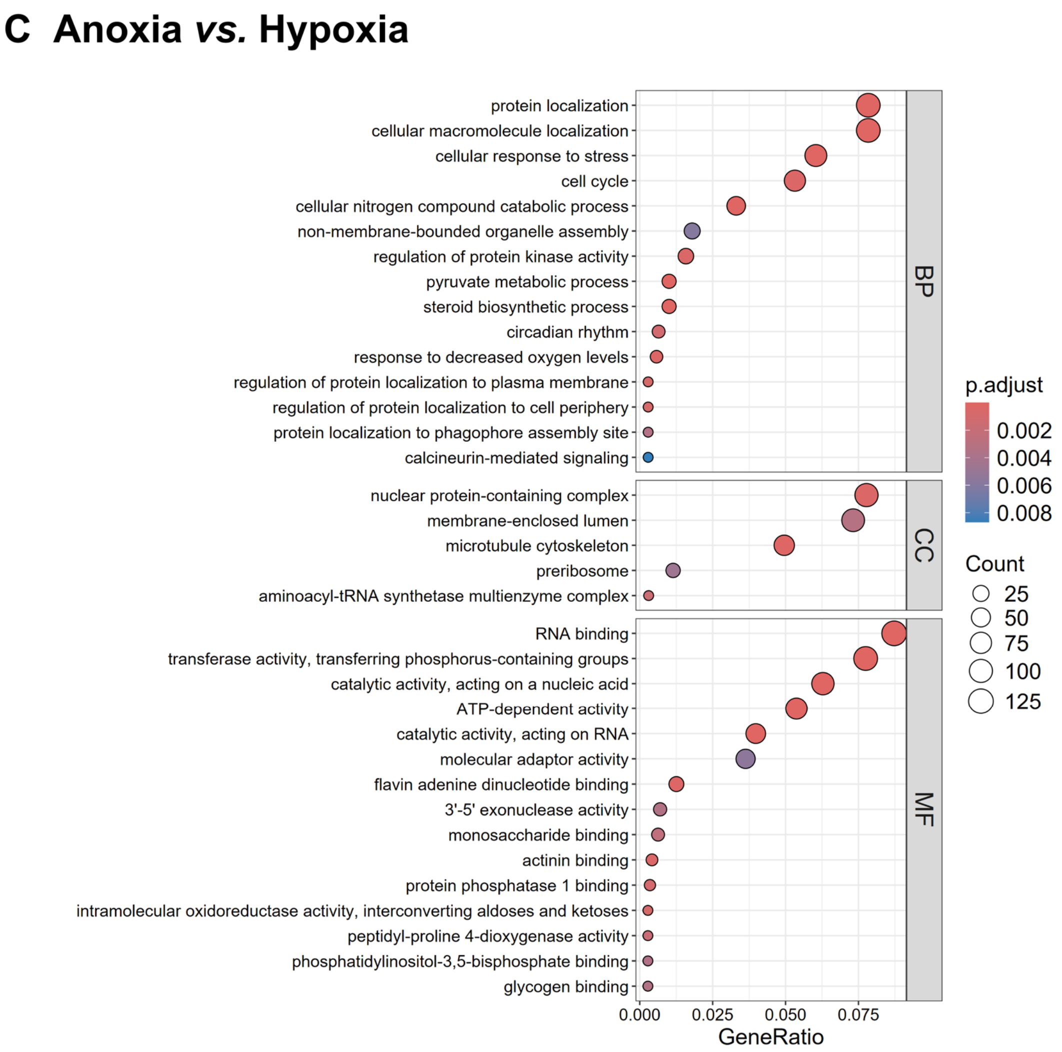

(A-C) Bubble charts showing the enriched pathways across the three GO categories. Redundant pathways were removed based on semantic similarity. The bubble size reflects the gene ratio, representing the proportion of DEGs in relation to the total genes assigned to a specific GO pathway. The color indicates the significance of the enrichment (adjusted P-values).

Figure S3. Kyoto Encyclopedia of Genes and Genomes (KEGG) enrichment analysis

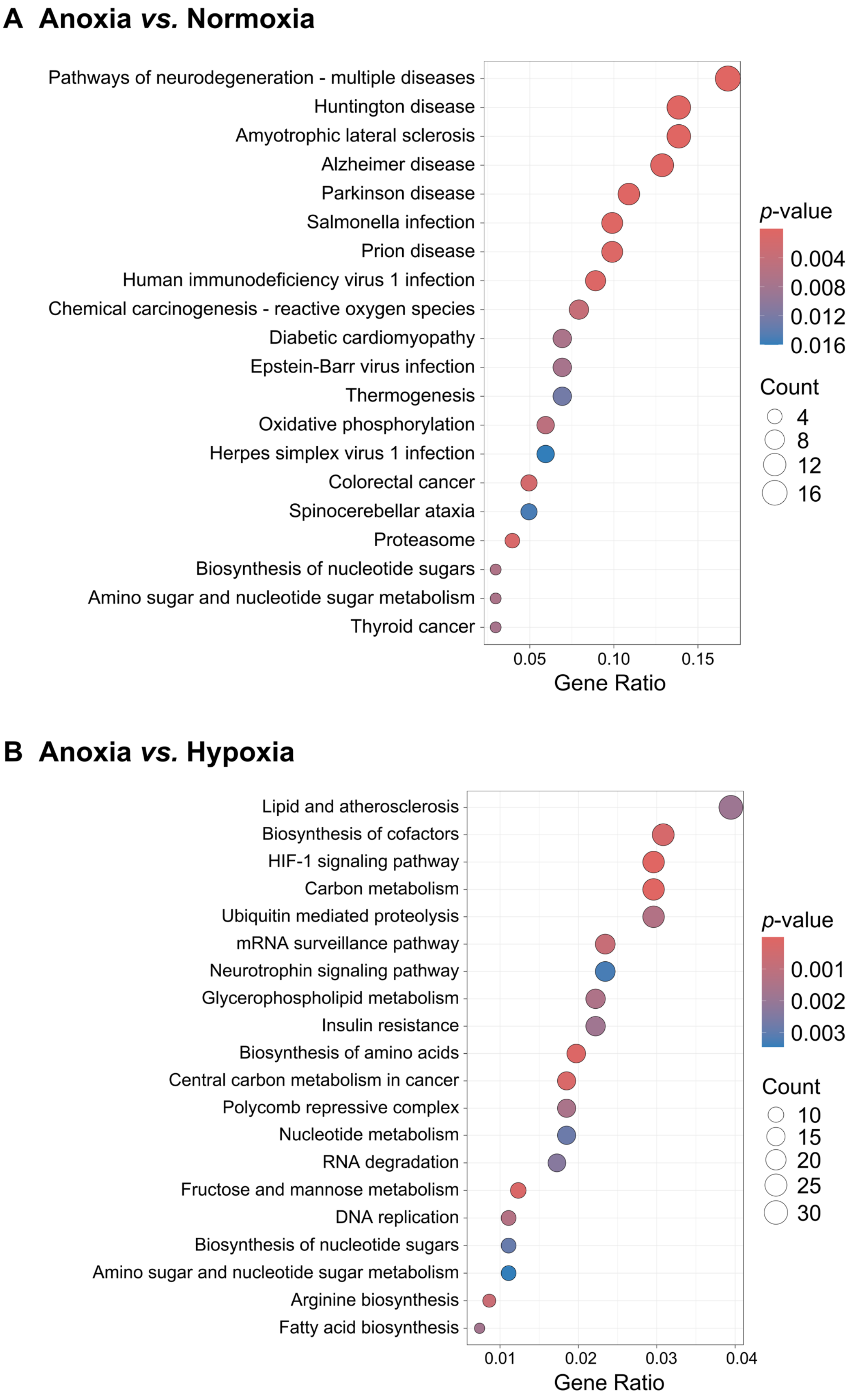

(A and B) Dot plot showing KEGG enrichment analysis in the two groups. The size of the dots represents the number of genes annotated to KEGG pathways. The colour from blue to red represents the significance of the enrichment. Gene ratio represents the ratio of the number of differentially expressed genes (DEGs) annotated to KEGG pathways to the total number of DEGs.

Figure S4. Identification of hub genes from the PPI network

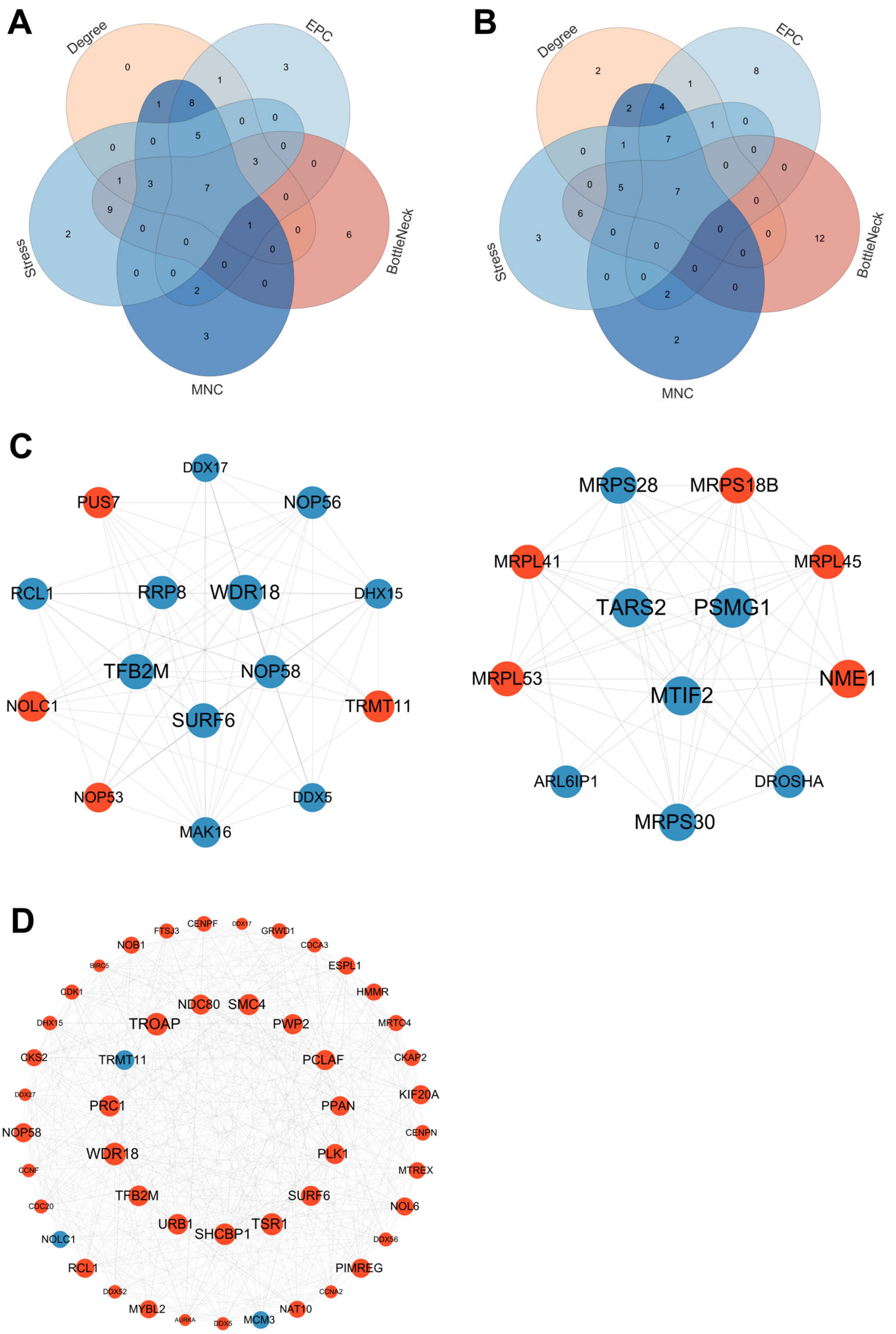

(A) Venn diagram of anoxia vs. normoxia. (B) Venn diagram of anoxia vs. hypoxia. Venn diagram showing the overlap of the top 30 hub genes identified using 5 different CytoHubba algorithms (Degree, EPC, Bottleneck, MNC, and Stress). The numbers in the figure represent the quantity of genes identified. (C) PPI network diagrams of hypoxia vs. normoxia. (D) PPI network diagrams of anoxia vs. hypoxia. PPI network visualisation of key genes constructed using STRING and Cytoscape. Red nodes represent upregulated genes, blue nodes represent downregulated genes.
